# Prediction of the Chemoreflex Gain by Common Clinical Variables in Heart Failure

**DOI:** 10.1371/journal.pone.0153510

**Published:** 2016-04-21

**Authors:** Gianluca Mirizzi, Alberto Giannoni, Andrea Ripoli, Giovanni Iudice, Francesca Bramanti, Michele Emdin, Claudio Passino

**Affiliations:** 1 Department of Cardiology and Cardiovascular Medicine, Fondazione Toscana G. Monasterio, Pisa, Italy; 2 Scuola Superiore Sant’Anna, Pisa, Italy; Universita degli Studi di Napoli Federico II, ITALY

## Abstract

**Background:**

Peripheral and central chemoreflex sensitivity, assessed by the hypoxic or hypercapnic ventilatory response (HVR and HCVR, respectively), is enhanced in heart failure (HF) patients, is involved in the pathophysiology of the disease, and is under investigation as a potential therapeutic target. Chemoreflex sensitivity assessment is however demanding and, therefore, not easily applicable in the clinical setting. We aimed at evaluating whether common clinical variables, broadly obtained by routine clinical and instrumental evaluation, could predict increased HVR and HCVR.

**Methods and results:**

191 patients with systolic HF (left ventricular ejection fraction—LVEF—<50%) underwent chemoreflex assessment by rebreathing technique to assess HVR and HCVR. All patients underwent clinical and neurohormonal evaluation, comprising: echocardiogram, cardiopulmonary exercise test (CPET), daytime cardiorespiratory monitoring for breathing pattern evaluation. Regarding HVR, multivariate penalized logistic regression, Bayesian Model Averaging (BMA) logistic regression and random forest analysis identified, as predictors, the presence of periodic breathing and increased slope of the relation between ventilation and carbon dioxide production (VE/VCO_2_) during exercise. Again, the above-mentioned statistical tools identified as HCVR predictors plasma levels of N-terminal fragment of proBNP and VE/VCO_2_ slope.

**Conclusions:**

In HF patients, the simple assessment of breathing pattern, alongside with ventilatory efficiency during exercise and natriuretic peptides levels identifies a subset of patients presenting with increased chemoreflex sensitivity to either hypoxia or hypercapnia.

## Introduction

Although modern therapies have improved the natural history of chronic heart failure (HF), mainly by tackling neurohormonal activation, the prognosis of HF is dismal [1,2,3] justifying the search for novel therapeutic targets in HF.

Chemoreflex sensitivity (CS) represents a major determinant of neurohormonal imbalance in HF, being associated with reduced baroreflex sensitivity [4,5], heightened sympathetic outflow and periodic breathing (PB) [6,5,7]. Far from being an innocent bystander, CS has been acknowledged as an independent prognosticator in HF, mainly due to a detrimental effect on the arrhythmic profile leading to cardiac mortality [8,9]. Specifically, CS impacted on prognosis mainly by increasing arrhythmic events and cardiac mortality, especially when both CS to hypoxia and hypercapnia were heightened (four-years survival 49%) compared to those with normal CS (survival 100%) [9].

Nowadays, a chemoreflex modulation strategy is plausible [10], according to a growing number of animal studies [11,12,13] demonstrating its feasibility and benefits. Indeed, in a ischaemic HF model in rats, carotid body denervation reduced the central pre-sympathetic neuronal activation, normalized indexes of sympathetic outflow and baroreflex sensitivity, and reduced the incidence of apnoea; there was also a time-dependent reduction in cardiac remodelling, deterioration of left ventricle ejection fraction, and cardiac arrhythmias; these modifications, most importantly, led to an amelioration in survival [13]. The feasibility of carotid body ablation and its autonomic effects in humans were recently confirmed by a pilot trial in a patient with chronic heart failure [14]. Hence, the possibility of feedback reflex modulation is in sight and the need of simple diagnostic tools for their evaluation is pressing.

CS is measured by specific tests, assessing the ventilatory response to either hypoxia or hypercapnia, in order to calculate the hypoxic ventilatory response (HVR) and hypercapnic ventilatory response (HCVR), respectively [15]. However, several limitations (need of dedicated instrumentations, physician supervision, patients’ discomfort) have hampered its clinical spread, which is currently limited to the research environment. However, in light of the previous consideration, it is clear that we need to implement on the clinical ground the evaluation of feedback control for a better risk stratification and follow-up.

Therefore, we sought to evaluate whether common clinical variables, broadly obtained by routine clinical and instrumental evaluation, could predict the presence of increased CS to hypoxia and hypercapnia in a population of systolic HF patients.

## Materials and Methods

From 2003 to 2011, we identified 191 consecutive HF patients from our outpatient clinic with echocardiographic evidence of impaired left ventricular systolic function (LVEF <50%) excluding those with: NYHA class IV, acute coronary syndrome within 6 months before examination, severe renal dysfunction (i.e. creatinine clearance < 35 ml/min), pulmonary disease (vital capacity and total lung capacity < 50% of predicted value; FEV1 [forced expiratory volume in 1 s] <50% of predicted value; and FEV1/FVC [forced vital capacity] <70%), and treatment with morphine or derivates, theophylline, oxygen, benzodiazepines or acetazolamide. All patients agreed participating in the study. They were on stable (i.e. >1 month) guideline-recommended treatment, with restriction of water/sodium intake. The study design included a standard clinical evaluation and: (1) the evaluation of chemosensitivity to hypoxia and to hypercapnia by assessing the individual HVR and HCVR; (2) neurohormonal evaluation; (3) echocardiography; (4) cardiopulmonary exercise testing (CPET) and (5) 20-min daytime polygraphy for the assessment of diurnal PB. The entire protocol was completed for each patient within 3 days.

The investigation was carried out in accordance with the Declaration of Helsinki of the World Medical Association, and has been approved by the local Ethics Committee "Comitato Etico di Area Vasta Nord-Ovest", Pisa, Italy. Patients provided written informed consent.

### Chemosensitivity assessment

**Fig 1:**
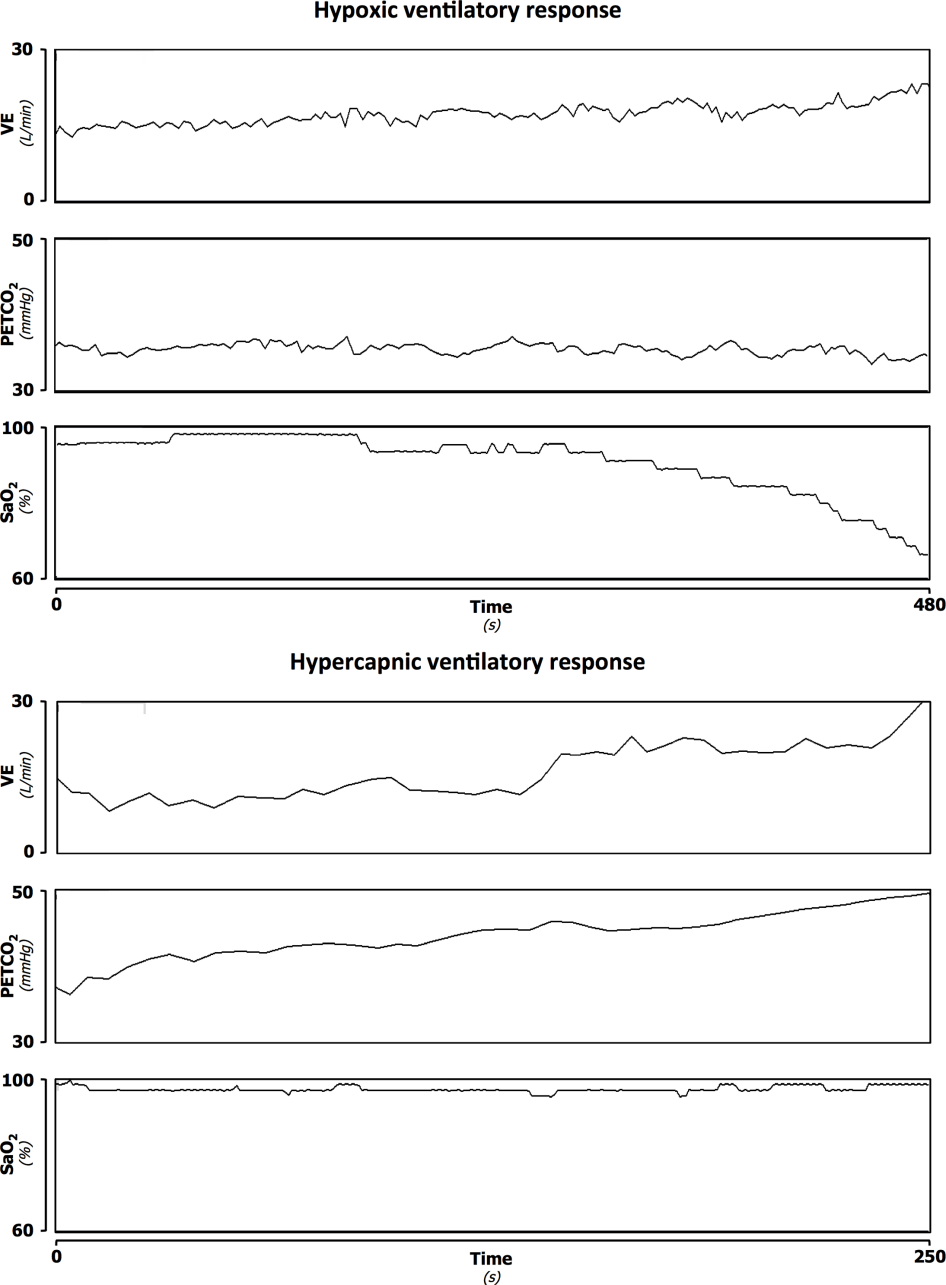
Example of an hypoxic normocapnic (upper panel) and an hypercapnic normoxic (lower panel) trial. In each panel are represented the time-dependent variation of minute ventilation (VE), end-tidal carbon dioxide production (PETCO_2_) and arterial oxygen saturation (SaO_2_) During the hypoxic trial, SaO2 diminish while PETCO_2_ remains constant due to correction by a scrubbing circuit. During the hypercapnic trial, SaO2 remains constant due to constant addition of O2 to the circuit.

**Fig 2:**
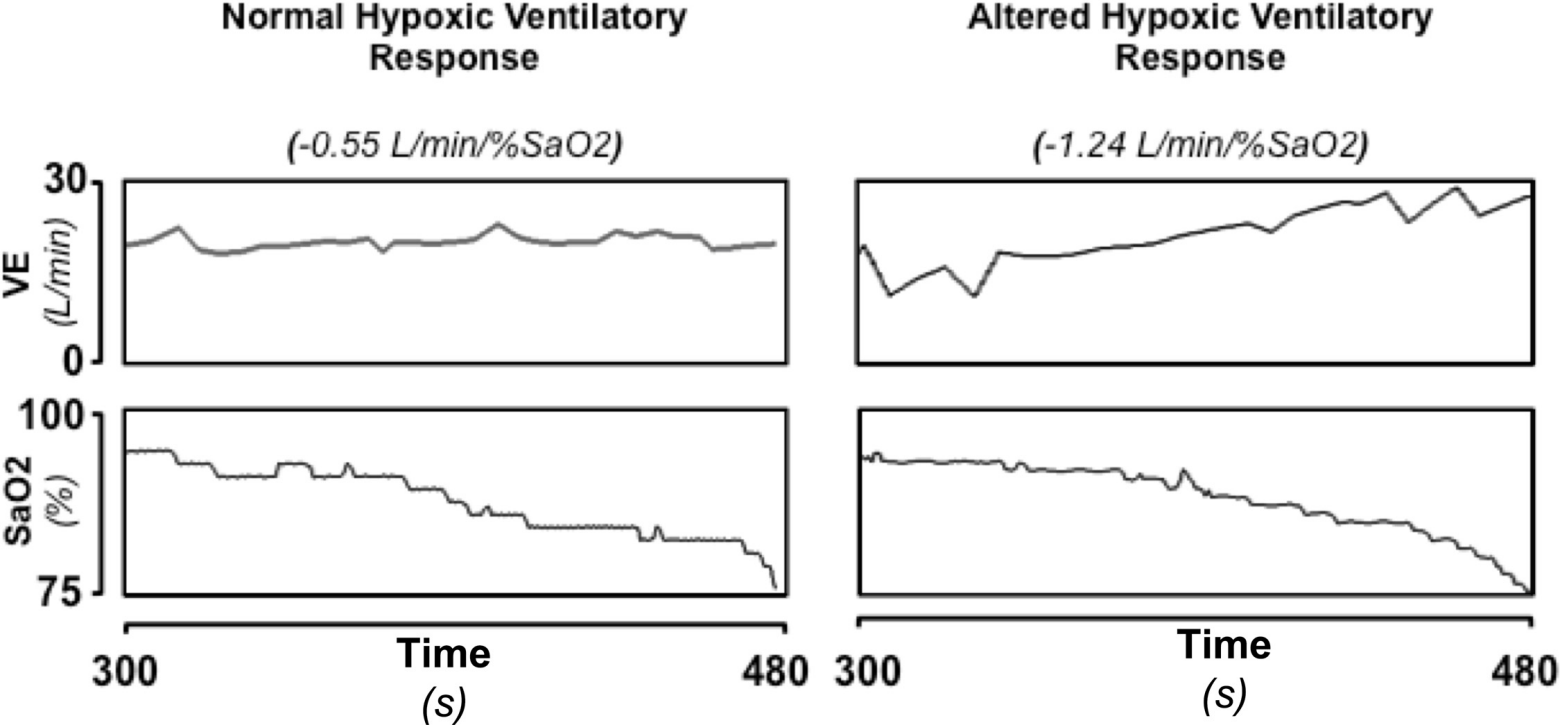
Example of a normal (left) and abnormal (right) hypoxic normocapnic trial. In each panel are represented the time-dependent variation of minute ventilation (VE), end-tidal carbon dioxide production (PETCO_2_) and arterial oxygen saturation (SaO_2_) During the hypoxic trial, SaO2 diminish while PETCO_2_ remains constant due to correction by a scrubbing circuit.

Chemosensitivity was assessed using the rebreathing technique [7]. Subjects were examined in standardized conditions, in a quiet room at a comfortable temperature, while seated and connected to a rebreathing circuit through a mouthpiece. They were not allowed to smoke, or drink alcohol or caffeine-containing beverages in the 12 h preceding the study. ECG, airway flow and respiratory gases were recorded continuously through a breath-by-breath gas analyser (Vmax; Sensormedics), and oxygen saturation was recorded through a pulse oximeter (SET® Radical; Masimo). A 4-min baseline recording was performed during spontaneous breathing. The mean SaO2 (arterial oxygen saturation) and end- tidal CO2 during this recording were assumed as subject resting values. During the progressive isocapnic hypoxia trial (from resting SaO2 values to 70–80%, according to individual tolerance), end-tidal CO2 was maintained at a baseline value by passing a portion of the expired air into a scrubbing circuit before returning it to a 5 litre rebreathing bag. Conversely, during the progressive normoxic hypercapnic trial (from resting end-tidal CO2 values until 50 mmHg or an increase ≥10 mmHg from the basal values, according to individual tolerance), inspired arterial partial pressure of oxygen was kept at the baseline value by adding oxygen to the circuit. The two trials were performed in a random order. Examples of hypoxic normocapnic and hypercapnic normoxic trials are shown in Fig 1; examples of a normal and an altered hypoxic normocapnic trial are shown in Fig 2. All signals were digitized online (500 sample/s; National Instruments) and analysed to derive respiratory rate, breath-to-breath VT (tidal volume) and VE (minute ventilation), as well as SaO_2_ and end-tidal pressure of CO_2_. HVR was expressed by the linear regression slope between VE and SaO_2_ during a hypoxic-normocapnic trial, while HCVR by the linear regression slope between VE and the end-tidal CO_2_ during the hypercapnic-normoxic trial.

### Neurohormonal evaluation, CPET, echocardiographic study, daytime cardiorespiratory recording

Plasma N-terminal pro-B-type natriuretic peptide (NT-proBNP), catecholamine and aldosterone levels, plasma renin activity (PRA) were assayed as previously described in details [16].

Patients underwent a symptom-limited CPET on a bicycle ergometer according to a ramp protocol to achieve maximal workload in 10 ± 2 minutes (Vmax; Sensormedics). Peak oxygen consumption (peak-VO_2_) was determined as the highest value at peak exercise over a 20-s average; ventilatory efficiency was estimated from the VE/VCO_2_ slope (calculated as the slope of the linear relationship between ventilation (VE) and carbon dioxide production (VCO_2_) from 1 min after the beginning of the loaded exercise and the end of the isocapnic buffering period. Echocardiography was assessed according to guidelines recommendations [17]. All CPETs and echocardiographic studies were performed by physicians blinded to the chemosensitivity tests results.

As previously described [18], all subjects, while awake and spontaneously breathing in a supine position, underwent a 20-min recording including two-leads ECG, chest wall and abdominal movements by electrical inductance, oronasal airflow by CO_2_ signal (Cosmoplus®; Novametrics) and SaO_2_ (Pulse Oxymeter Pulsox-7; Minolta). Breathing pattern was evaluated visually observing the traces of nasal flow and respiratory movements; apnoea was defined as the cessation of inspiratory flow for at least 10 s, whereas hypopnoea was defined as a flow reduction (>50%) lasting 10s or more and associated with at least a 4% decrease in SaO_2_ [19]. Apnoea and hypopnoea were considered as central or obstructive by the absence or presence of ribcage and abdominal excursions, respectively. We only considered central apnoea for our analysis.

### Statistical analysis

Data are reported as mean ± standard deviation for normally distributed variables; otherwise, they are expressed as median (interquartile range, IR). A p ≤0.05 was considered statistically significant.

Target variables (HVR and HCVR) were dichotomized using the previously described cut-off values (increased chemosensitivity if HVR>0.77 l·min^-1^·%SaO_2_^-1^ and HCVR>0.79 l·min^-1^·mmHg^-1^) [7]. Association between target variables and covariates was studied constructing a univariate logistic regression model. Covariates that resulted significantly associated at univariate analysis (p<0.10) were used to create a multivariate model. Owing to the high number of variables in comparison with the number of cases, we identified covariates with two distinct methods: the penalized approach and the Bayesian Model Averaging (BMA) approach [20]. This statistical approach has the clear advantage of bootstrapping multiple models by randomly selecting variables from the overall data set and assessing pre- and post-hoc probabilities of prediction to evaluate the best possible models (and predictors), according to Bayes' principle, thus providing an adequate stability to the analysis. The output of this approach are a set of variables with a given odds ratio, thus identifying variables for which each unit increase (for example, each percent unit increase in LVEF) produces an increase or decrease of the probability of having an heightened chemoreflex sensitivity (that is, respectively, "adverse" or "protective" variables).

Parallel to this approach, in order to catch the effects of non-linear interaction between variables that are missed by conventional statistical methods, we also performed a random forest (RF) analysis, as described elsewhere [21]. Briefly, RF is an ensemble classifier build up from a set of decision trees created from a subset of training data (2/3 of original data), randomly constructed by choosing variables and samples (bootstrapping). The pre-specified rules with which the trees are assembled decides whether to split the tree (knot) or terminate it (terminal knot) at a value deemed to be the best split point for each variable. RF analysis gives the accuracy in the prediction of an observation in a sample; furthermore, it allows to rank a set of variables based on their importance in predicting the observation. For continuous variables it does not give a specific cut-off but takes into account the repercussions of the variable on the accuracy of the prediction (i.e., its increase whenever the variable is taken into account). Also for this reason, this approach has the advantage with respect to conventional statistics to avoid overfitting models and to be less sensitive to outliers. Analyses were performed using the R open-source statistical software.

## Results

### Patients' characteristics

Patients’ general characteristics are enlisted in Table 1. Overall, HVR and HCVR were increased in 34% and 56% of patients, respectively. Patients were mainly males (83%), aged (62, SD 14 years), with balanced ischemic/non ischemic aetiology (48/52%, respectively) and with a moderate to severe LV dysfunction at echocardiography (mean left ventricular ejection fraction [LVEF] 30, SD 8%). At CPET, they showed a reduced functional capacity (mean peak VO_2_/kg: 13.8, SD 6.3 ml·min^-1^·kg^-1^) and reduced ventilatory efficiency (VE/VCO_2_ slope: 37, SD 9); roughly half of the population presented with diurnal periodic breathing (PB) (48%). Neurohormonal activation was modest, with only augmented levels of plasma NT-proBNP (1117, IR 86–6851 ng/l). All patients were on optimal medical therapy. Patients with enhanced HVR as compared with those with normal chemosensitivity, showed increased ventilatory inefficiency, plasma levels of noradrenaline and gamma-glutamyltransferase (GGT), increased right ventricular (RV) dimensions. Those with increased HCVR as compared with patients with normal chemosensitivity were instead older, had lower LVEF, reached a lower maximal workload and showed increased ventilatory inefficiency; they also showed increased plasma levels of NT-proBNP, noradrenaline, GGT and greater right ventricular (RV) diameter (data not shown).

**Table 1:** Clinical characteristics of the HF patient population.

Variables	Population n = 191
Age, y	62±14
Males, %	83
Body Mass Index (BMI), kg/m^2^	27.0±4.2
Ischemic Etiology, %	48
NYHA Class I-II, %	72
Periodic Breathing/ Cheyne-Stokes respiration, %	47/24
Hypoxic Ventilatory Response (HVR), l*min^-1^*%SaO2^-1^	0.5 (0.2–1.2)
Hypercapnic Ventilatory Response (HCVR), l*min^-1^*mmHg^-1^	0.85 (0.2–2.1)
Left Ventricular Ejection Fraction (LVEF), %	30±8
End Systolic Left Ventricular Diameter, mm	51±10
End Diastolic Left Ventricular Diameter, mm	62±8
Sinus Rhythm/Atrial Fibrillation, %	73/27
Workload, W	80 (40–174)
Peak Oxygen Consumption, (pVO_2_), ml*min^-1^*kg^-1^	13.8±6.2
VE/VCO_2_ slope	37±9
Serum Creatinine, mg/dl	1.2±0.4
Norepinephrine, pg/ml	435 (141–1033)
Plasma Renin Activity, ng*ml^-1^* h^-1^	1.2 (0.2–11.3)
NT-proBNP, pg/ml	1117 (86–6851)
Plasma Aldosterone, pg/ml	140 (31–399)
Beta-Blockers, %	84
ACE-inhibitors or ARBs, %	77
Aldosterore Receptors Blockers, %	56
Diuretics, %	79

ACE: angiotensin-converting enzyme; ARB: angiotensin receptor blockers; NYHA, New York Class Association; NT-proBNP: N-type fragment of proBNP; VE/VCO_2_ slope: slope of the relationship of CO_2_ production and ventilatory response during exercise.

### Hypoxic chemosensitivity: univariate and multivariate analysis

At univariate analysis, HVR was associated with the following 9 covariates: LVEF, RV dimension, left atrial dimensions, baseline ventilation, VE/VCO_2_ slope, NT-proBNP levels, GGT levels, presence of atrial fibrillation and PB.

**Fig 3:**
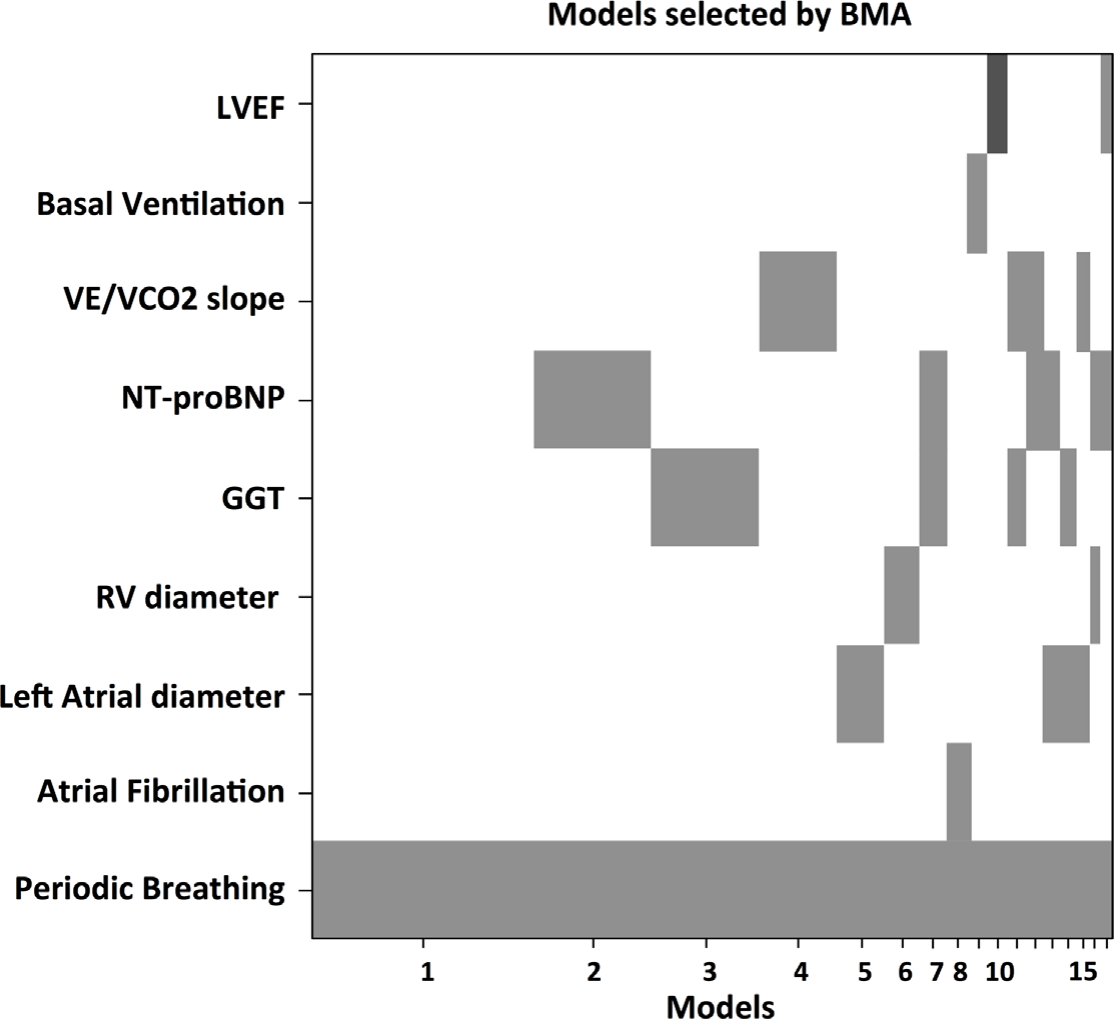
Bayesian Model Averaging analysis of HVR predictors. Dark grey: protective; light grey: adverse. GGT: gamma-glutamyltransferase; LVEF: left ventricular ejection fraction; NT-proBNP: N-type fragment of pro-brain natriuretic peptide; PB: periodic breathing; RV: right ventricle; VE/VCO2 slope: slope of the relationship of CO2 production and ventilatory response during exercise.

At multivariate penalized analysis (Table 2), HVR was associated with left atrial dimensions (odds ratio [OR] 1.012, confidence interval [C.I.] 1.005–1.148), VE/VCO_2_ slope (OR 1.01, 95th C.I. 1.007–1.131), NT-proBNP levels (OR 1.007, C.I. 1.003–1.081 for each 100 units increase), GGT levels (OR 1.004, C.I. 1.001–1.153), and, more strongly with the presence of PB (OR 5.401, CI 3.712–12.655). BMA showed the same findings, with the presence of PB being the strongest variable associated with altered HVR (OR 7.91, Fig 3).

**Table 2:** Penalized multivariate analysis of HVR predictors.

Variable	OR (95% I.R.)
VE/VCO_2_ slope	1.010 [1.007–1.131]
NT-proBNP	1.007 [1.003–1.081]
GGT	1.004 [1.001–1.153]
Left atrial diameter	1.012 [1.005–1.148]
Presence of PB	5.401 [3.712–12.655]

GGT: gamma glutamyltransferase; NT-proBNP: N-type fragment of proBNP; PB: periodic breathing;VE/VCO_2_ slope: slope of the relationship of CO_2_ production and ventilatory response during exercise.

**Fig 4:**
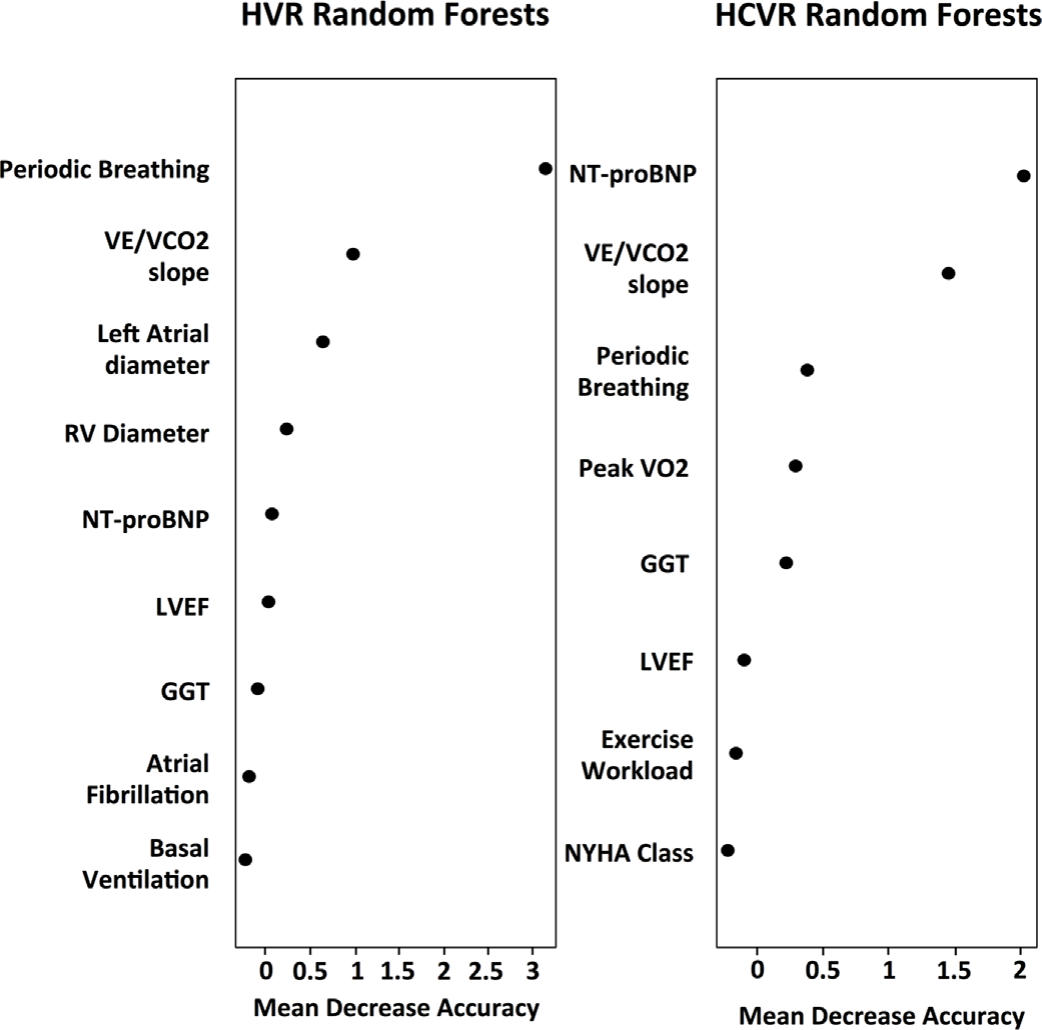
Random forests analysis for HVR and HCVR predictors. Variable importance plots displaying predictors from random forests (RF) analysis of HVR and HCVR. Variable importance is assessed by the mean decrease accuracy. Mean decrease accuracy is the normalized difference of classification accuracy when the data for that variable is included as observed, and the classification accuracy when the values of the variable have been randomly permuted. Higher values of mean decrease in accuracy indicate variables that are more important to the classification. Acronyms same as in Fig 3.

Random Forest analysis warranted an out of bag accuracy of 71%, identifying the presence of PB and increased VE/VCO_2_ slope as covariate associated with HVR (Fig 4); in particular, the presence of PB conferred an increase in prediction accuracy of 3.13%.

### Hypercapnic chemosensitivity: univariate and multivariate analysis

At univariate analysis, HCVR was associated with the following 8 covariates: LVEF, NYHA class, peak VO_2_/kg, VEVCO_2_ slope, exercise workload, NT-proBNP levels, GGT levels and PB.

**Fig 5:**
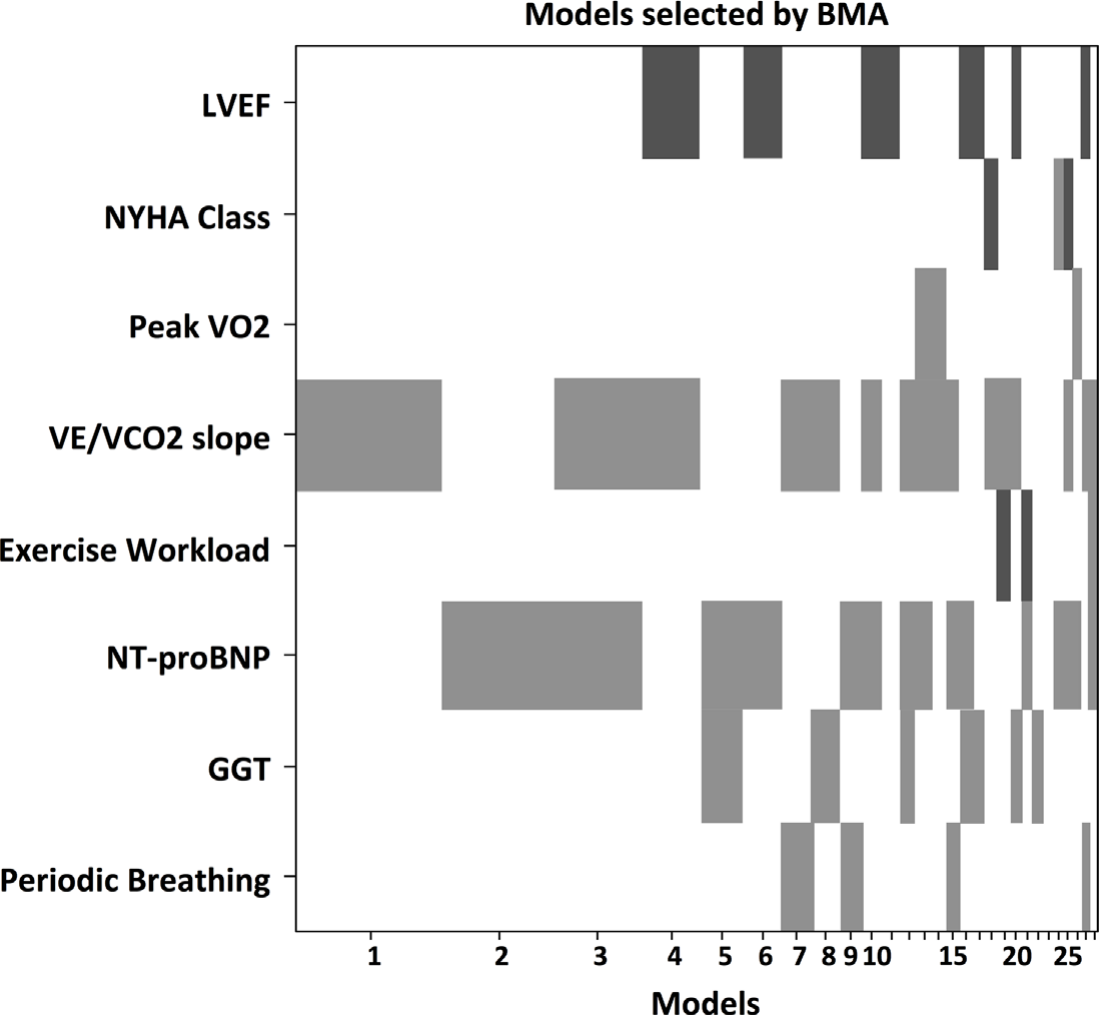
Bayesian Model Averaging analysis of HCVR predictors. Colour coding and acronyms same as Fig 2.

At penalized multivariate analysis, HCVR was associated with LVEF (OR 0.976, 95th C.I. 0.647–0.985), VE/VCO_2_ slope (OR 1.028, C.I. 1.009–1.250), NT-proBNP (OR 1.009, C.I. 1.002–1.059 for each 100 units increase), GGT (OR 1.002, C.I. 1.0007–1.357), PB (OR 1.096, C.I. 1.005–1.766) (Table 3). BMA found a weak evidence for VE/VCO_2_ slope (OR 1.04) and NT-proBNP (OR 1.01 for each 100 units increase, Fig 5).

**Table 3:** Penalized multivariate analysis of HCVR predictors.

Variable	OR (95% I.R.)
LVEF	0.976 [0.647–0.985]
NT-proBNP	1.009 [1.002–1.059]
GGT	1.002 [1.007–1.357]
VE/VCO_2_ slope	1.028 [1.009–1.250]
Presence of PB	1.096 [1.005–1.766]

GGT: gamma-glutamyltransferase; LVEF: left ventricular ejection fraction; NT-proBNP: N-type fragment of pro-brain natriuretic peptide; PB: periodic breathing; RV: right ventricle; VE/VCO2 slope: slope of the relationship of CO2 production and ventilatory response during exercise.

Random Forest analysis warranted an out of bag accuracy of 58%, identifying the presence of NT-proBNP and VE/VCO_2_ slope as covariate associated with HCVR (Fig 4); in particular, the presence of NT-proBNP conferred an increase in prediction accuracy of 1.95%, while VE/VCO2 slope of 1.37%.

## Discussion

In this study, we assessed the clinical predictors of altered chemosensitivity to hypoxia and hypercapnia in a cohort of systolic HF patients suggesting that chemoreflex, a critical pathophysiological determinant of HF progression and prognosis, may be predicted by using few clinical parameters easily obtainable and commonly assessed in whichever HF clinic: a blood withdrawal (NT-proBNP), a cardiopulmonary test (VE/VCO_2_ slope) and a short-term cardiorespiratory monitoring (diurnal PB). In fact, they correctly predicted HVR and HCVR in 70% and 60% of cases, respectively.

### The clinical relevance of assessing the CS in HF

Growing evidences support the notion that increased CS to hypoxia [8] and hypercapnia [9] act as pernicious players in the HF pathophysiological background. Beyond its acknowledged role as the mechanism underlying PB [6,8,9,22], increased CS has been associated with worse symptoms, lower performance during exercise, higher sympathetic activity and increased arrhythmic risk [7,8, 23]. These pathophysiological alterations lead to a significant increase in the risk of cardiac death and arrhythmic events in HF. In the study of Ponikowski et al [8], 3-year survival in those with heightened peripheral CS was 41% vs. 77% in those with normal one. When assessed in a well characterized and on guideline-directed medical therapy comprehensive of beta-blockers (86%) and implantable-cardioverter device/cardiac resynchronization therapy, increased CS still maintains its prognostic value, especally when both peripheral and central CS are augmented (4-year survival 49% vs. 100% in those with normal CS) [9]. It is also significant that the studies assessing the prognostic significance of altered CS to hypoxia used different methods to assess it (transient hypoxic method by Ponikowski and rebreathing technique by Giannoni); while no specific comparison between the two methods have not been undertaken, the clinical significance of both might suggest that the biological phenomenon, and not the specific measure to estimate it, is of relevance for the clinician.

Animal studies have also demonstrated that CS modulation reverses the above-mentioned negative consequences and improves survival [13,24]. In fact, in an ischaemic rat model of chronic HF carotid body ablation reduced pre-sympathetic activation, overall sympathetic outflow, normalized baroreflex activation and attenuated cardiac remodelling and LEVF reduction; these translated in an increase in survival from 45 to 85%. If a beneficial prognostic effect should be confirmed in humans after the demonstration of the safety, feasibility and tolerability of carotid body surgical resection [14], this could lead to the opening of a strategy of chemoreflex modulation in HF.

The application to a broad population of these new developments requires an adequate evaluation of CS. Since now this has been hampered by the difficult spread of the chemoreflex tests, due to technical issues (need of trained physicians, time-consuming test execution and analysis) and patient discomfort, potentially making suboptimal both risk stratification and treatment approach in HF patients, especially in the fore coming new era of the feedback control therapy.

Even if the implementation of a widespread assessment of CS is still the goal to be pursued, a convenient strategy may be the identification of a set of common clinical parameters whose alteration could suggest the presence of altered CS to hypoxia or hypercapnia and prompt the evaluation by specialized laboratories which deal with respiratory derangements in HF and their diagnosis. It seems thus judicious to include as much as possible candidates with altered CS while avoiding their exclusion (i.e. prefer sensitivity to specificity); from this point of view, for example, the identification of 11% of patients (i.e., one out of ten) with PB but without increased HVR might represent a reasonable trade-off to avoid such under-diagnosis while sparing, on the other hand, chemoreflex evaluation in a consistent portion of patients (53%).

### The first clinical model for prediction of CS in HF

Since now, mathematical models [25,26,27] and clinical studies [28] have focused on the possibility to predict PB using the chemoreflex as one of the possible contributors and not as the real target, raising attention to the epiphenomenon and not to the mechanism behind it.

In this respect, to our knowledge, this is the first attempt to predict CS to both stimuli (hypoxia and hypercapnia) starting from a comprehensive and large cohort of patients fully characterized and under guideline-recommended therapy. In fact, in a small series of HF patients (n = 34) a similar approach have been recently confined to the exclusive prediction of peripheral CS, with NT-proBNP identified as the only independent predictor of HVR: however, the small number of patients recruited and the type of statistics performed limits the strength of study results [29]. The rigorous statistical model developed in this study has allowed us to identify 3 main clinical predictors of CS, namely PB, VE/VCO_2_ slope and NT-proBNP, with the possibility to infer the hypoxic and hypercapnic ventilatory response straight off, without actually performing any test.

In our study, only using PB and VE/VCO_2_ slope it is possible to closely predict HVR, with an out of bag accuracy of 71% from RF analysis. The strongest variable in this model is represented by PB, which conferred an increase in prediction accuracy of more than 3 times. This relationship has been already highlighted in several previous reports [30,31]. Normalization of peripheral CS, by administration of dihydrocodeine or exposition to hyperoxia in humans [32] and by carotid body monolateral or bilateral cryoablation in animals [24] resulted in normalization of breathing pattern. Our study supports these previous findings. Hence, in clinical practice, recognition of PB, much easier to be evaluated in respect to CS, could act as a red flag for identifying patients with altered HVR.

Our data suggest VE/VCO2 slope as another contributor in the prediction of HVR. HVR is a measure of the peripheral chemosensitivity. It is known that peripheral chemoreceptors also account for the ventilatory response to carbon dioxide increase (about 20% of the global response), so a relationship between HVR and VE/VCO2 slope is likely. Moreover, during effort the contribution of peripheral chemoreceptors may be increased [33].

VE/VCO_2_ slope, in fact, also allowed the prediction of the HCVR in our HF patient cohort, replicating the observation made by previous reports in HF patients [7,32]. In fact, VEVCO_2_ slope might be considered another possible way of assessing the ventilatory response to CO_2_, with CO_2_ released by the working muscles and not provided from outside. It must be noted that a contributor to VE/VCO2 slope is given by increased pulmonary dead space, especially in more advanced disease states [34]; however, it was recently associated, in an HF population, to chronic obstructive pulmonary disease comorbidity [35], which we excluded as entry criterion. The emergence of VE/VCO2 as a predictor of increased chemoreflex sensitivity, despite the exclusion of dead space as a cofactor, further stresses the importance of the relation between the two parameter, not always recognized in the adequate manner.

NT-proBNP also plays a role in the prediction of HCVR, as already found in a different cohort by our group [7]. Two reasons of this observation could be made: first, in an animal study performed on pacing induced HF rabbit, a reduction of peripheral chemoreceptor perfusion by carotid occlusion similar to the one obtained with cardiac output reduction (and potentially expressed by higher level of NT-proBNP), was associated with increased peripheral chemoreceptors, which are also responsive to CO_2_, thus contributing to HCVR [36,37]. Second, an increase in the diastolic filling pressure (again sensed by the natriuretic peptide system) with stimulation of pulmonary J receptors, may justify a medullary-mediated augmentation of both peripheral and central chemoreceptor reflexes. Generally, NT-proBNP levels could synthesize the multiple influences of the various axes (renin angiotensin aldosterone systems, adrenergic systems) on both peripheral and central chemoreceptors [23,38,39].

On the whole, the absolute predictive power of the model is weaker for HCVR than for HVR (predictive accuracy respectively 58 vs. 71%), likely owing to the different physiological complexity of the two reflexes. HVR relies only on the response of the peripheral chemoreceptor to O2 tension reduction, while HCVR express the response of both peripheral (20%) and central chemoreceptors (80%) to rise in CO_2_. The interaction between peripheral and central chemoreceptors, as well as the multiplicity of different anatomic locations and physiologic responses of central chemoreceptors [40] adds to the complexity and justifies the findings.

### Study limitations

Despite the limited number of patients recruited, this is one of the largest cohorts of HF patients with complete evaluation of peripheral and central chemoreflexes. Despite the lack of control normal population, the results are consistent because of the statistical approach, that used an internal group (1/3 of the population) as learning subset and the rest of the population as testing subset. Moreover, the number of patients with altered CS is relatively high, allowing the performance of meaningful statistical analysis. Nevertheless, some patients (23%, n = 44) showed combined increased CS; we decided to evaluate this subgroup of patients singularly for CS, and not as a separate subgroup, because the number of patients could hardly permit a separate statistical evaluation of adequate consistency and the relative small number of individuals should not affect statistical output.

The population evaluated was composed exclusively of patients with systolic HF; no patients with HF and preserved ejection fraction were evaluated. Diastolic dysfunction could influence CS status acting on pulmonary J receptors and enhancing ventilatory responses. In dog models of congestive HF, inflating a balloon in the left atrium, mimicking the increases in left ventricular end-diastolic pressure increases encountered clinically, causes hyperventilation and increase in loop gain of the ventilatory response to CO2 [41]. It must also be recalled that in recent series of systolic HF patients, neither LVEF nor diastolic function were associated with increased pulmonary artery pressure, while were chemoreflex status and the presence of central apnoeas [42] thus highlighting the independent influences of feedback loops on hemodynamics and principally on that of the pulmonary circulation.

We choose to perform multiple multivariate statistical analysis in order to avoid the possibility of overfitting models, which cannot however be excluded. The concordance between findings of standard statistical methods and computational iterative statistics gives strength to the overall results, even if reproducibility of the sets of variables identified is an issue to be verified. Testing patients under conditions of normocapnic or normoxic conditions during respectively HVR and HCVR could provide less reliable results due to individual variability in the response to CO2 or O2. However, while this is relevant in a physiological scenario, it is less relevant in a clinical scenario like ours, because global response to hypoxia or hypercapnia is what patients with heart failure develop in their daily living, for example during cycles of apnoea; moreover, these same stimuli have been demonstrated to increase the risk of events in the same patients [9].

## Conclusion and clinical perspectives

In HF patients, the assessment of breathing pattern, alongside with ventilatory efficiency during exercise and natriuretic peptides levels, helps to identify the subset of patients with increased hypoxic or hypercapnic chemosensitivity. Using these parameters in daily clinical practice may allow the identification of patients with altered CS and prompt the standard evaluation of chemosensitivity; this approach could help widely including the chemoreflex status in the risk stratification of HF patients. This information might be used to contribute to the setup of rational follow-up strategies and to build up a tailored pharmacological/non-pharmacological approach specifically directed to the chemoreflex target.
